# On Points Focusing Entropy

**DOI:** 10.3390/e20020128

**Published:** 2018-02-16

**Authors:** Ewa Korczak-Kubiak, Anna Loranty, Ryszard J. Pawlak

**Affiliations:** Faculty of Mathematics and Computer Science, ódź University, Banacha 22, 90-238 ódź, Poland

**Keywords:** nonautonomous (autonomous) dynamical system, topological entropy, (asymptotical) focal entropy point, disturbation, m-dimensional manifold, 54C70, 37A35, 37B40, 58C30, 26A18

## Abstract

In the paper, we consider local aspects of the entropy of nonautonomous dynamical systems. For this purpose, we introduce the notion of a (asymptotical) focal entropy point. The notion of entropy appeared as a result of practical needs concerning thermodynamics and the problem of information flow, and it is connected with the complexity of a system. The definition adopted in the paper specifies the notions that express the complexity of a system around certain points (the complexity of the system is the same as its complexity around these points), and moreover, the complexity of a system around such points does not depend on the behavior of the system in other parts of its domain. Any periodic system “acting” in the closed unit interval has an asymptotical focal entropy point, which justifies wide interest in these issues. In the paper, we examine the problems of the distortions of a system and the approximation of an autonomous system by a nonautonomous one, in the context of having a (asymptotical) focal entropy point. It is shown that even a slight modification of a system may lead to the arising of the respective focal entropy points.

## 1. Introduction and Preliminaries

In many papers dealing with dynamical systems, their strong relation to difference equations is pointed out (see [1]), which gives the possibilities of their wide applications in many fields of knowledge, including economics, biology, information flow or physics [2,3,4,5,6,7]. Among the problems connected with “dynamical systems with discrete time observations”, a special role is played by the entropy of these systems, which may be treated as a “measure” of the complexity of a dynamical system. This notion was introduced with respect to the issues connected with thermodynamics and the problem of “information loss” (more details on this topic can be found in [8]). At the beginning, the notion of entropy was related to the measure theory. Later, there appeared the notion of topological entropy introduced by R. Adler, A. Konheim and J. McAndrew [9], and next, an equivalent definition for metric spaces was formulated [10,11]. It is worth mentioning that in the further stage of research, the definition of topological entropy for discontinuous functions was also studied [12]. The considerations mentioned concerned autonomous systems. Later, still, there appeared results regarding the entropy of nonautonomous dynamical systems. We will base our investigations, among others, on [13]. In general, the notion of entropy concerns a global property of dynamical systems. However, research connected for example with stability points or non-wandering points, as well as the analysis of various examples of functions lead to the conclusion that it is also purposeful to examine local aspects of entropy and points around which the entropy is “focused” in some sense, e.g., [14,15]. Simultaneously, the example presented in [16] (p. 1118) shows that it is intentional to assume that the essence of a point “focusing” entropy should be connected with the behavior of functions only (exclusively) around this point or the value of functions at this point (sometimes, the fact that a point is a full entropy point [15] is decisively influenced by the behavior of a function “far away” from that point). For that reason, a new approach to this problem was introduced in [16]. All the above-mentioned papers concerned autonomous systems. In this paper, we will refer to these issues as nonautonomous dynamical systems. Our considerations will be mainly connected with the periodicity of the examined systems. Such kind of investigations are frequently connected in the literature with such systems (e.g., [6,13,17]). It is caused by the connections of such systems with periodic difference equations (it is well signalled in [3]).

Throughout the paper, the symbol N will stand for the set of all positive integers and $N_{0}=N\cup \left\{0\right\}$. Moreover, (X,ρ) will denote a compact metric space. The closure, the interior and the cardinality of a set A⊂X will be denoted by cl(A), int(A) and #(A), respectively. For any function f:X→X and sets A,B⊂X, the symbols f↾A and $A\underset{f}{\to }B$ mean the restriction of *f* to *A* and B⊂f(A), respectively.

The symbol ${\mathrm{FIX}}_{X}\left(x_{0}\right)$ will denote the family of all self-maps defined on *X* such that the point $x_{0}$ is their fixed point, and the symbol FIX(f) will stand for the set of all fixed points of a function *f*. Moreover, for any functions f,g:X→X, let us adopt the following notation ≠(f,g)={x∈X:f(x)≠g(x)}.

Let (X,ϱ) be a metric space and ${\left\{K_{n}\right\}}_{n\in N}$ be a sequence of nonempty closed subsets of *X*. We shall say that the sequence ${\left\{K_{n}\right\}}_{n\in N}$ has the extension property if for any i,j∈N and any continuous function $\varphi :A\to K_{j}$, where $A\subset K_{i}$ is a closed set, one can find a continuous function $\psi :K_{i}\to K_{j}$, which is an extension of φ, i.e., ψ↾A=φ. Obviously, if for example $X=R^{n}$ and $K_{n}$ are cubes, then this fact follows from the generalizations of the classical Tietze theorem.

Following [13], by a nonautonomous dynamical system on *X* (NDS), we will mean any sequence of functions $f_{1,\infty }={\left\{f_{i}\right\}}_{i\in N}$ such that $f_{i}:X\to X$. If $f_{i}=f$ for i∈N, then we call the system autonomous and denote it by (f). For n∈N, let $f_{n,\infty }=\left\{f_{n},f_{n+1},\cdots \right\}$ and $f_{1,\infty }^{n}={\left\{f_{(i-1)\cdot n+1}^{n}\right\}}_{i\in N}$, where $f_{i}^{n}=f_{n+i-1}\circ f_{n+i-2}\circ \cdots \circ f_{i+1}\circ f_{i}$. Moreover, let $f_{i}^{0}=f_{i}^{-0}=\mathrm{id}$ (where id is the identity function) and $f_{i}^{-n}=f_{i}^{-1}\circ f_{i+1}^{-1}\circ \cdots \circ f_{i+(n-1)}^{-1}$ for any i,n∈N (the last notation will be applied to sets, so we do not assume that these maps are invertible). If f:X→X is a function, then for any n∈N, the symbol $f^{n}$ will denote the *n*-th iteration of *f*, i.e., $f^{n}=f\circ f^{n-1}$ and $f^{0}=\mathrm{id}$.

We say that a dynamical system $f_{1,\infty }$ is periodic with a period *n* if $f_{k}=f_{k\mathrm{mod}n}$, if kmodn≠0 and $f_{k}=f_{n}$ otherwise. Moreover, we say that $x_{0}$ is a periodic point with a period *n* of an NDS $f_{1,\infty }$ if $x_{0}$ is a fixed point of an NDS $f_{1,\infty }^{n}$, i.e., $f_{(i-1)\cdot n+1}^{n}\left(x_{0}\right)=x_{0}$ for any i∈N.

If *M* is a matrix, then the trace of *M* will be denoted by tr(M). Let ${\left\{M_{n}\right\}}_{n\in N}$ be a sequence of square matrices of the same degree *t*. Then, for any k∈N, we will consider $\prod_{i=1}^{k}M_{i}=M_{1}\cdot M_{2}\cdot \cdots \cdot M_{k}$.

In [13] was introduced a Bowen-like definition of entropy for an NDS consisting of continuous functions. This definition was expanded for systems consisting of arbitrary functions in the paper [8]. We will briefly review that notion.

Let n∈N and ε>0. A set E⊂X is called (n,ε)-separated if for any two distinct points x,y∈E, there exists j∈{0,⋯,n−1} such that $\rho (f_{1}^{j}\left(x\right),f_{1}^{j}\left(y\right))>\varepsilon$. If Y⊂X, then *E* is (n,ε)-separated in *Y* if it satisfies the above condition and E⊂Y. Let $s_{n}\left(f_{1,\infty },Y,\varepsilon \right)$ denote the maximal cardinality of the (n,ε)-separated set in *Y*. Then, the entropy of a system $f_{1,\infty }$ on *Y* is the number:

$$h\left(f_{1,\infty },Y\right)=\lim_{\varepsilon \to 0}\underset{n\to \infty }{lim sup}\frac{1}{n}\log s_{n}\left(f_{1,\infty },Y,\varepsilon \right).$$

If Y=X, then we write briefly $h(f_{1,\infty })$ instead of $h(f_{1,\infty },X)$. Moreover, if we consider an autonomous system (f), then the entropy of this system will be denoted by h(f,Y) and h(f), respectively. By the entropy of a function *f*, we will mean the entropy of a respective autonomous system (f).

Now, we will signal, in the form of lemmas, basic facts that will be used in the further part of the paper. Reasoning similar to that in the proofs of Lemma 4.3 and 4.5 [13] allows proving the following result concerning the entropy of an NDS consisting of not necessarily continuous functions.

**Lemma** **1.** *Let $f_{1,\infty }$ be a dynamical system. Then, for any n≥1, we have:*

$$h\left(f_{1,\infty }^{n}\right)\leq n\cdot h\left(f_{1,\infty }\right).$$

**Lemma** **2.** Let $f_{1,\infty }$ be a dynamical system on X. For any 1≤i≤j<∞, we have $h\left(f_{i,\infty }\right)\leq h\left(f_{j,\infty }\right)$.

In the case of NDS, entropy does not always fully reflect the complexity of a system (see, e.g., the considerations in [13], p. 216). Therefore, in [13] was introduced a new notion of asymptotical entropy, which, with respect to autonomous systems, coincides with the classical entropy.

An asymptotical entropy of a dynamical system $f_{1,\infty }$ is the number $h^{*}\left(f_{1,\infty }\right)$ defined as follows: $h^{*}\left(f_{1,\infty }\right)=\lim_{n\to \infty }h\left(f_{n,\infty }\right)$. The existence of such a limit follows from Lemma 2. Moreover, Lemma 2 allows concluding that $h\left(f_{1,\infty }\right)\leq h^{*}\left(f_{1,\infty }\right)$. It is worth adding that the inequality from Lemma 2 is not true for entropy on subsets of the space, so the asymptotical entropy of a system on a set Y⊂X is defined as the following upper limit:

$$h^{*}\left(f_{1,\infty },Y\right)=\underset{n\to \infty }{lim sup}h\left(f_{n,\infty },Y\right).$$

Our terminology and notations related to *m*-dimensional manifolds will coincide with those of [18]. An *m*-dimensional manifold with a boundary is a nonempty compact metric space (M,d) such that every point q∈M has a neighborhood *U* that is homeomorphic (via a transformation called the chart on *U*) to an open subset of the *m*-dimensional upper half space $H^{m}=\left\{\left(x_{1},...,x_{m}\right)\in R^{m}:x_{m}\geq 0\right\}$. Since any open ball in $R^{m}$ is homeomorphic to some open subset of $H^{m}$, an *m*-dimensional topological manifold is an *m*-dimensional topological manifold with a boundary (with an empty boundary). Therefore, in this paper, we will consider only *m*-dimensional topological manifolds with a boundary.

If M is a nonempty *m*-dimensional manifold with a boundary, a point that belongs to the inverse image of $\mathrm{int}\left(H^{m}\right)=\left\{\left(x_{1},...,x_{m}\right)\in R^{m}:x_{m}>0\right\}$ under some chart is called an interior point of M. The set of all interior points of a manifold M will be denoted by Int(M). The symbol $B_{M}$ will stand for the set of all closed submanifolds M of M (i.e., M⊂M is a closed manifold) such that the dimensions of M and M are the same.

We shall say that an NDS $\left(f_{1,\infty }\right)$ of functions defined on M is irreducible at $x_{0}$ if for n∈N, a function $f_{1}^{n}$ is irreducible at $x_{0}$, i.e., for any open neighborhood *U* of $x_{0}$, there exists a point $y_{0}\in \mathrm{Int}\left(M\right)\cap U$ such that $f_{1}^{n}\left(x_{0}\right)\neq f_{1}^{n}\left(y_{0}\right)$.

## 2. Focal Entropy Points of NDS

Now, we will introduce the notion of a focal entropy point of NDS, having in mind the general assumption: the fact that a given point is a focal entropy point means that the complexity of the system in any neighborhood of this point is the same as the complexity of the whole system and does not depend on the behavior of functions around other points.

Let A be a family of nonempty subsets of *X* such that each nonempty open set contains some element of A. In view of the considerations presented in this paper, from now on, we will assume that A contains the family of all closed sets of cardinality continuum.

Put $\Theta \left(A\right)=\{\left(A_{1},\cdots ,A_{m}\right):A_{1},\cdots ,A_{m}\in A,m\in N,\mathrm{cl}\left(A_{i}\right)\cap \mathrm{cl}\left(A_{j}\right)=\emptyset \;\mathrm{for}\;i\neq j\}$.

Let $A=\left(A_{1},\cdots ,A_{m}\right)\in \Theta \left(A\right)$ and n∈N. Set $M_{f_{n}}\left(A\right)={\left[a_{i,j}^{f_{n}}\right]}_{i,j\leq m}$, where:

$$a_{i,j}^{f_{n}}=\left\{\begin{array}{cc} 1 & \;\mathrm{if}\;A_{i}\underset{f_{n}}{\to }A_{j},\\ 0 & \;\mathrm{if}\;A_{j}\backslash f_{n}\left(A_{i}\right)\neq \emptyset . \end{array}\right.$$

Moreover, for k∈N, a system $\left(f_{1,\infty }\right)$ and $A=\left(A_{1},\cdots ,A_{m}\right)\in \Theta \left(A\right)$, let:

(1) $$M_{f_{1,\infty }}^{k}\left(A\right)=\prod_{n=1}^{k}M_{f_{n}}\left(A\right):={\left[a_{i,j}^{k}\right]}_{i,j\leq m}.$$

The entropy of $f_{1,\infty }$ with respect to the sequence A is the following number:

$$H_{f_{1,\infty }}\left(A\right)=\left\{\begin{array}{cc} \underset{k\to \infty }{lim sup}\frac{1}{k}\log\mathrm{tr}\left(M_{f_{1,\infty }}^{k}\left(A\right)\right) & \;\mathrm{if}\;\mathrm{tr}(M_{f_{1,\infty }}^{k}\left(A\right))>0,\\ 0 & \;\mathrm{if}\;\mathrm{tr}(M_{f_{1,\infty }}^{k}\left(A\right))=0. \end{array}\right.$$

The process of computing the entropy of a system with respect to a sequence of sets may be simplified by introducing the notion of a path. Let k∈N. For a *k*-path connected with the sequence A and with the dynamical system $\left(f_{1,\infty }\right)$, we call each sequence of sets $\left(A_{p_{1}},A_{p_{2}},\cdots ,A_{p_{k}}\right)$ such that $p_{i}\in \left\{1,\cdots ,m\right\}$ for i=1,⋯,k and:

$$A_{p_{1}}\underset{f_{1}}{⟶}A_{p_{2}}\underset{f_{2}}{⟶}A_{p_{3}}\underset{f_{3}}{⟶}\cdots \underset{f_{k-2}}{⟶}A_{p_{k-1}}\underset{f_{k-1}}{⟶}A_{p_{k}}.$$

The sets $A_{p_{1}},A_{p_{2}},\cdots ,A_{p_{k}}$ are called the nodes of the path. If no confusion can arise, we will write simply *k*-path. We say that a point $x_{0}\in A_{p_{1}}$ is connected with a *k*-path $\left(A_{p_{1}},A_{p_{2}},\cdots ,A_{p_{k}}\right)$ if $f_{1}^{i}\left(x_{0}\right)\in A_{p_{i+1}}$ for i=1,⋯,k−1. It is easy to see that such a point exists for any path.

One can easily notice that the entry $a_{i,j}^{k}$, where i,j∈{1,⋯,m}, of the matrix (1) is equal to the number of (k+1)-paths connected with the sequence A and the NDS $f_{1,\infty }$ such that the set $A_{i}$ is the first node of the path and the set $A_{j}$ is its last node. Consequently, $\mathrm{tr}(M_{f_{1,\infty }}^{k}\left(A\right))$ is equal to the number of (k+1)-paths connected with the sequence A and the NDS $f_{1,\infty }$ such that the set $A_{i}$ is simultaneously the first and the last node of the path, for i=1,⋯,m.

Now, let us state the theorem, which will allow introducing the next steps of the definition.

**Theorem** **1.** *Let $f_{1,\infty }$ be an NDS, $A=\left(A_{1},\cdots ,A_{m}\right)\in \Theta \left(A\right)$ and n∈N. Then:*

$$H_{f_{1,\infty }^{n}}\left(A\right)\leq h\left(f_{1,\infty }^{n}\right)\leq n\cdot h\left(f_{1,\infty }\right).$$

**Proof.**  The second inequality follows from Lemma 1, so it is sufficient to show the first inequality. Suppose, contrary to our claim, that there exists a real number α such that:

(2) $$h\left(f_{1,\infty }^{n}\right)<\alpha <H_{f_{1,\infty }^{n}}\left(A\right).$$

It is obvious that α>0 and $H_{f_{1,\infty }^{n}}\left(A\right)>0$. According to our assumptions connected with the family Θ(A), we have $\varepsilon_{A}=\frac{1}{2}\min\left\{\rho \left(\mathrm{cl}\left(A_{i}\right),\mathrm{cl}\left(A_{j}\right)\right):i,j\in \left\{1,\cdots ,m\right\}\wedge i\neq j\right\}>0.$ Taking into account (2), we obtain that there exists an increasing sequence of positive integers ${\left\{k_{s}\right\}}_{s\in N}$ such that:

(3) $$\frac{1}{k_{s}}\log\mathrm{tr}\left(M_{f_{1,\infty }^{n}}^{k_{s}}\left(A\right)\right)>\alpha \;\mathrm{for}\;s=1,2,\cdots$$

Clearly, $a_{1,1}^{k_{s}},a_{2,2}^{k_{s}},\cdots ,a_{m,m}^{k_{s}}$ are successive entries of the main diagonal of the matrix $M_{f_{1,\infty }^{n}}^{k_{s}}\left(A\right)$, for any s∈N. By (3), one can conclude that for any s∈N, we have $N_{k_{s}}=\left\{i\in \left\{1,\cdots ,m\right\}:a_{i,i}^{k_{s}}>0\right\}\neq \emptyset$. For any s∈N and i∈{1,⋯,m}, the number of $\left(k_{s}+1\right)$-paths of the form $A_{i}\underset{f_{1}^{n}}{⟶}A_{p_{1}}\underset{f_{n+1}^{n}}{⟶}A_{p_{2}}\underset{f_{2n+1}^{n}}{⟶}\cdots \underset{f_{(k_{s}-2)n+1}^{n}}{⟶}A_{p_{k_{s}-1}}\underset{f_{(k_{s}-1)n+1}^{n}}{⟶}A_{i},$ where $p_{w}\in \left\{1,\cdots ,m\right\}$ for $w=1,\cdots ,k_{s}-1$, is equal to $a_{i,i}^{k_{s}}$. For any s∈N and $i\in N_{k_{s}}$, let $\beta_{i}^{k_{s}}$ denote the set of all $\left(k_{s}+1\right)$-paths whose first and last node is $A_{i}$. Obviously $\#\left(\beta_{i}^{k_{s}}\right)=a_{i,i}^{k_{s}}$. Therefore, let $\beta_{i}^{k_{s}}=\left\{B_{i,1}^{k_{s}},B_{i,2}^{k_{s}},\cdots ,B_{i,a_{i,i}^{k_{s}}}^{k_{s}}\right\}$. For any s∈N, $i\in N_{k_{s}}$ and $j\in \{1,\cdots ,a_{i,i}^{k_{s}}\}$, let $b_{i,j}^{k_{s}}$ be a point connected with the path $B_{i,j}^{k_{s}}$.

Put $\Delta \left(k_{s}\right)=\left\{b_{i,j}^{k_{s}}:i\in N_{k_{s}}\wedge j\in \left\{1,\cdots ,a_{i,i}^{k_{s}}\right\}\right\}$ for s∈N. It is easily seen that $b_{i,j}^{k_{s}}\in A_{i}$ for s∈N, $i\in N_{k_{s}}$ and $j\in \{1,\cdots ,a_{i,i}^{k_{s}}\}$. Thus, if $i_{1},i_{2}\in N_{k_{s}}$ and $i_{1}\neq i_{2}$, then $b_{i_{1},j}^{k_{s}}\neq b_{i_{2},j}^{k_{s}}$. Moreover, if $j_{1},j_{2}\in \left\{1,\cdots ,a_{i,i}^{k_{s}}\right\}$ and $j_{1}\neq j_{2}$, then $b_{i,j_{1}}^{k_{s}}\neq b_{i,j_{2}}^{k_{s}}$. Thus, $\#\left(\Delta \left(k_{s}\right)\right)=\sum_{i\in N_{k_{s}}}\#\left(\beta_{i}^{k_{s}}\right)=\sum_{i\in N_{k_{s}}}a_{i,i}^{k_{s}}$, and finally, $\#\left(\Delta \left(k_{s}\right)\right)=\sum_{i=1}^{m}a_{i,i}^{k_{s}}$, because $a_{i,i}^{k_{s}}=0$ for $i\in \left\{1,\cdots ,m\right\}\backslash N_{k_{s}}$.

Let $b_{i_{1},j_{1}}^{k_{s}},b_{i_{2},j_{2}}^{k_{s}}$ be any distinct points of the set $\Delta (k_{s})$. If $i_{1}\neq i_{2}$, then $\rho \left(b_{i_{1},j_{1}}^{k_{s}},b_{i_{2},j_{2}}^{k_{s}}\right)\geq \rho \left(\mathrm{cl}\left(A_{i_{1}}\right),\mathrm{cl}\left(A_{i_{2}}\right)\right)>\varepsilon_{A}$. If $i_{1}=i_{2}=i$, then $j_{1}\neq j_{2}$. Thus, since $b_{i,j_{1}}^{k_{s}}$ is connected with the path $B_{i,j_{1}}^{k_{s}}=\left(A_{i,j_{1}},A_{p_{1},j_{1}},\cdots ,A_{p_{k_{s}-1},j_{1}},A_{i,j_{1}}\right)$ and $b_{i,j_{2}}^{k_{s}}$ is connected with the path $B_{i,j_{2}}^{k_{s}}=\left(A_{i,j_{2}},A_{p_{1},j_{2}},\cdots ,A_{p_{k_{s}-1},j_{2}},A_{i,j_{2}}\right)$ and $B_{i,j_{1}}^{k_{s}}\neq B_{i,j_{2}}^{k_{s}}$, so there exists $w_{0}\in \left\{1,\cdots ,k_{s}-1\right\}$ such that $A_{p_{w_{0}},j_{1}}\neq A_{p_{w_{0}},j_{2}}$ and $\rho \left(f^{w_{0}\cdot n}\left(b_{i,j_{1}}^{k_{s}}\right),f^{w_{0}\cdot n}\left(b_{i,j_{2}}^{k_{s}}\right)\right)\geq \rho \left(\mathrm{cl}\left(A_{p_{w_{0}},j_{1}}\right),\mathrm{cl}\left(A_{p_{w_{0}},j_{2}}\right)\right)>\varepsilon_{A}$. This gives that $\Delta (k_{s})$ is the $\left(k_{s},\varepsilon_{A}\right)$-separated set for the system $\left(f_{1,\infty }^{n}\right)$.

As a consequence, we obtain $s_{k_{s}}\left(f_{1,\infty }^{n},\varepsilon_{A}\right)\geq \#\left(\Delta \left(k_{s}\right)\right)=a_{1,1}^{k_{s}}+\cdots +a_{m,m}^{k_{s}}$. Let $\varepsilon \in (0,\varepsilon_{A})$. Thus, $\underset{l\to \infty }{lim sup}\frac{1}{l}\log s_{l}\left(f_{1,\infty }^{n},\varepsilon \right)\geq \underset{s\to \infty }{lim sup}\frac{1}{k_{s}}\log\sum_{i=1}^{m}a_{i,i}^{k_{s}}=\underset{s\to \infty }{lim sup}\frac{1}{k_{s}}\log\mathrm{tr}\left(M_{f_{1,\infty }^{n}}^{k_{s}}\left(A\right)\right)\geq \alpha$, and hence, $h\left(f_{1,\infty }^{n}\right)=\lim_{\varepsilon \to 0}\underset{l\to \infty }{lim sup}\frac{1}{l}\log s_{l}\left(f_{1,\infty }^{n},\varepsilon \right)\geq \alpha$, which contradicts (2). ☐

We continue the considerations leading to the definition of a focal entropy point. Let U⊂X be an open set. For $A=\left(A_{1},\cdots ,A_{m}\right)\in \Theta \left(A\right)$, the notation A⊂U will mean that $A_{i}\subset U$ for any i∈{1,⋯,m}. Let us adopt the following notation:

$$H\left(A,f_{1,\infty },U\right)=\sup\left\{\frac{1}{n}H_{f_{1,\infty }^{n}}\left(A\right):A\in \Theta \left(A\right)\wedge A\subset U\wedge n\in N\right\}.$$

Notice that on account of Theorem 1, for any open set *U*, we have:

(4) $$H\left(A,f_{1,\infty },U\right)\leq h\left(f_{1,\infty }\right).$$

Put:

$$d\left(A,f_{1,\infty },U\right)=\left\{\begin{array}{cc} \frac{H(A,f_{1,\infty },U)}{h(f_{1,\infty })} & \;\mathrm{if}\;h\left(f_{1,\infty }\right)\in \left(0,\infty \right),\\ 1 & \;\mathrm{if}\;H\left(A,f_{1,\infty },U\right)=\infty \;\mathrm{or}\;h\left(f_{1,\infty }\right)=0,\\ 0 & \;\mathrm{if}\;H\left(A,f_{1,\infty },U\right)\in \left[0,\infty \right)\;\mathrm{and}\;h\left(f_{1,\infty }\right)=\infty . \end{array}\right.$$

Using the last quantity, one can define the next one in the following way:

$$E\left(A,f_{1,\infty },x_{0}\right)=\inf\left\{d\left(A,f_{1,\infty },U\right):U\in O\left(x_{0}\right)\right\},$$

where $O(x_{0})$ denotes the family of all open sets containing $x_{0}$.

According to Theorem 1, we have $E(A,f_{1,\infty },x_{0})\leq 1$. If $E(A,f_{1,\infty },x_{0})=1$, then we say that a point $x_{0}\in X$ is a A-focal entropy point of a system $f_{1,\infty }$.

Notice that if a system $f_{1,\infty }$ is autonomous, i.e., $f_{i}=f$ for i∈N, then the definition of a A-focal entropy point of the system $f_{1,\infty }$ coincides with the definition introduced in [16].

If in the definition of the quantity $d(A,f_{1,\infty },U)$ we will replace an entropy $h(f_{1,\infty })$ with asymptotical entropy $h^{*}\left(f_{1,\infty }\right)$, then by defining in an analogous way as above, we will obtain the notion of a asymptotical A-focal entropy point of $f_{1,\infty }$. In such a case, we will use a star in the respective symbols: $d^{*}\left(A,f_{1,\infty },U\right)$, $E^{*}\left(A,f_{1,\infty },x_{0}\right)$. Therefore, we say that a point $x_{0}\in X$ is an asymptotical A-focal entropy point of a system $f_{1,\infty }$ if $E^{*}\left(A,f_{1,\infty },x_{0}\right)=1$.

It is easy to see that if $x_{0}$ is an asymptotical A-focal entropy point of a system $f_{1,\infty }$, then it is a A-focal entropy point of this system. Obviously, if $f_{1,\infty }$ is periodic, then the notions of an asymptotical A-entropy point of the system and of a A-focal entropy point of the system coincide.

The natural question arises whether there exist such points. The next theorem is a partial answer to this problem.

**Theorem** **2.** Let $f_{1,\infty }$ be a periodic dynamical system on [0,1] consisting of continuous functions. Then, there exists an asymptotical A-focal entropy point of the system $f_{1,\infty }$.

**Proof.**  Let *n* be a period of the system $f_{1,\infty }$. Put $f=f_{1}^{n}$ and $g_{1,\infty }=f_{1,\infty }^{n}$. Then, $g_{1,\infty }=\left(f\right)$. Hence, by Lemma 1, we obtain:

(5) $$h\left(g_{1,\infty }\right)=n\cdot h\left(f_{1,\infty }\right).$$

Moreover, notice that:

(6) $$g_{1,\infty }^{k}=f_{1,\infty }^{n\cdot k}\;\mathrm{for}\;k\in N.$$

By Corollary 4.5 [16], there exists a point $x_{0}\in \left[0,1\right]$, which is a A-focal entropy point of $g_{1,\infty }$.

We will show that $x_{0}$ is a A-focal entropy point of the system $f_{1,\infty }$. Let $U\in O(x_{0})$. It is easy to observe that $E(A,g_{1,\infty },x_{0})=1$ and consequently $d(A,g_{1,\infty },U)=1$. We need to consider the following cases (we omit the trivial case $h(g_{1,\infty })=0$):

(i) $H(A,g_{1,\infty },U)=\infty$. Thus, $\sup\{\frac{1}{k}H_{g_{1,\infty }^{k}}\left(A\right):A\in \Theta \left(A\right)\wedge A\subset U\wedge k\in N\}=\infty$. For any β>0, there exist $k_{\beta }\in N$ and $A_{\beta }\in \Theta \left(A\right)$ such that $A_{\beta }\subset U$ and $\frac{1}{k_{\beta }}H_{g_{1,\infty }^{k_{\beta }}}\left(A_{\beta }\right)>n\cdot \beta$. Obviously, by (6), we have $g_{1,\infty }^{k_{\beta }}=f_{1,\infty }^{n\cdot k_{\beta }}$, so $\frac{1}{k_{\beta }}H_{f_{1,\infty }^{n\cdot k_{\beta }}}\left(A_{\beta }\right)>n\cdot \beta$, and therefore, $\frac{1}{n\cdot k_{\beta }}H_{f_{1,\infty }^{n\cdot k_{\beta }}}\left(A_{\beta }\right)>\beta$. As a consequence, $\sup\{\frac{1}{s}H_{f_{1,\infty }^{s}}\left(A\right):A\in \Theta \left(A\right)\wedge A\subset U\wedge s\in N\}>\beta$. Hence and from arbitrariness β, we conclude that $\sup\{\frac{1}{s}H_{f_{1,\infty }^{s}}\left(A\right):A\in \Theta \left(A\right)\wedge A\subset U\wedge s\in N\}=\infty$, and consequently, $d(A,f_{1,\infty },U)=1$.
(ii) $h\left(g_{1,\infty }\right)\in \left(0,\infty \right)$ and $H\left(A,g_{1,\infty },U\right)=h\left(g_{1,\infty }\right)$. By (5), we obtain $h\left(f_{1,\infty }\right)\in \left(0,\infty \right)$. We have $h\left(g_{1,\infty }\right)=\sup\left\{\frac{1}{k}H_{g_{1,\infty }^{k}}\left(A\right):A\in \Theta \left(A\right)\wedge A\subset U\wedge k\in N\right\}$, so for any β>0, there exist $k_{\beta }\in N$ and $A_{\beta }\in \Theta \left(A\right)$ such that $A_{\beta }\subset U$ and $\frac{1}{k_{\beta }}H_{g_{1,\infty }^{k_{\beta }}}\left(A_{\beta }\right)>h\left(g_{1,\infty }\right)-n\cdot \beta$. Clearly, by (6), we may infer that $g_{1,\infty }^{k_{\beta }}=f_{1,\infty }^{n\cdot k_{\beta }}$, so $\frac{1}{k_{\beta }}H_{f_{1,\infty }^{n\cdot k_{\beta }}}\left(A_{\beta }\right)>h\left(g_{1,\infty }\right)-n\cdot \beta$. By use of (5), we get $\frac{1}{n\cdot k_{\beta }}H_{f_{1,\infty }^{n\cdot k_{\beta }}}\left(A_{\beta }\right)>h\left(f_{1,\infty }\right)-\beta$. Finally, we have shown that for any β>0, there exist $l_{\beta }=n\cdot k_{\beta }\in N$ and $A_{\beta }\in \Theta \left(A\right)$ such that $A_{\beta }\subset U$ and $\frac{1}{l_{\beta }}H_{f_{1,\infty }^{l_{\beta }}}\left(A_{\beta }\right)>h\left(f_{1,\infty }\right)-\beta$, so:
  (7) $$\sup\left\{\frac{1}{k}H_{f_{1,\infty }^{k}}\left(A\right):A\in \Theta \left(A\right)\wedge A\subset U\wedge k\in N\right\}\geq h\left(f_{1,\infty }\right).$$
  Moreover, according to (4), we have:
  (8) $$H\left(A,f_{1,\infty },U\right)\leq h\left(f_{1,\infty }\right).$$
  Finally, (7) and (8) give $H\left(A,f_{1,\infty },U\right)=\sup\left\{\frac{1}{k}H_{f_{1,\infty }^{k}}\left(A\right):A\in \Theta \left(A\right)\wedge A\subset U\wedge k\in N\right\}=h\left(f_{1,\infty }\right)$. Thus, $d(A,f_{1,\infty },U)=1$.

Since *U* was chosen arbitrarily, we obtain $E(A,f_{1,\infty },x_{0})=1$, so $x_{0}$ is a A-focal entropy point of $f_{1,\infty }$, and simultaneously, it is its asymptotical A-focal entropy point because this system is periodic. ☐

## 3. Disturbance and Approximation

In various considerations connected with autonomous and nonautonomous dynamical systems, a special role is played by fixed points of the systems (e.g., stable points [6]). It is not difficult to find an example showing that a fixed point of NDS need not be its focal entropy point. On the other hand, a given NDS can be approximated or disturbed by entering new functions into it. In each of these operations, it is important to do it by means of functions that are close to the base NDS and belong to the common structure. This leads in a natural way to distinguishing equivalence classes.

Let $f,g\in {\mathrm{FIX}}_{X}\left(x_{0}\right)$ and ε>0. In the set ${\mathrm{FIX}}_{X}\left(x_{0}\right)$, we will define the following relation:

(9) $$f\frac{\varepsilon }{x_{0}}g\Leftrightarrow \neq \left(f,g\right),f\left(\neq \left(f,g\right)\right),g\left(\neq \left(f,g\right)\right)\subset B\left(x_{0},\varepsilon \right),$$

where $B(x_{0},\varepsilon )$ is an open ball with radius ε and center $x_{0}$. It is not difficult to show that for the fixed ε>0 and $x_{0}\in X$, the relation (9) is an equivalence relation in ${\mathrm{FIX}}_{X}\left(x_{0}\right)$.

The symbol ${\left[f\right]}_{x_{0}}^{\varepsilon }$ will stand for the equivalence class of $f\in {\mathrm{FIX}}_{X}\left(x_{0}\right)$ under the relation $\frac{\varepsilon }{x_{0}}$.

In this paper are mainly examined periodic dynamical systems, so it is natural to consider periodic disruptions called disturbances. The idea of the disturbance is introducing, in equal periods of time, a function belonging to the equivalence class generated by the iteration of functions lying between successive disturbance periods.

Let $f_{1,\infty }$ be a periodic NDS with a period $k_{0}\in N$, and let ε>0. We say that $T_{1,\infty }^{\varepsilon }$ is a periodic ε-disturbance of $f_{1,\infty }$ if there exists a continuous function ψ such that:

(PD1) $T_{1,\infty }^{\varepsilon }=\left\{f_{1},f_{2},\cdots ,f_{k_{0}},\psi ,f_{1},f_{2},\cdots ,f_{k_{0}},\psi ,\cdots \right\},$
(PD2) $\psi \in {\left[f_{1}^{k_{0}}\right]}_{x_{0}}^{\varepsilon }$.

The next theorem shows that a periodic dynamical system may be periodically disturbed by means of a function belonging to an earlier defined equivalence class (with arbitrary small ε) in such a way that a periodic point of the system becomes its asymptotical A-focal entropy point.

**Theorem** **3.** Let $f_{1,\infty }$ be a periodic dynamical system on M consisting of continuous functions such that $x_{0}\in M$ is a periodic point of this NDS and $f_{1,\infty }$ is irreducible at $x_{0}$. For any ε>0, there exists a system $T_{1,\infty }^{\varepsilon }$ that is a periodic ε-disturbance of $f_{1,\infty }$ such that $x_{0}$ is an asymptotical A-focal entropy point of $T_{1,\infty }^{\varepsilon }$.

**Proof.**  Let $m_{0}$ be a period of $f_{1,\infty }$ and $m_{1}$ be a period of $x_{0}$. Put $n_{0}=m_{0}\cdot m_{1}$. It follows immediately that $n_{0}$ is both a period of $f_{1,\infty }$ and of $x_{0}$. Let ε>0 and ${\left\{M_{n}\right\}}_{n=0}^{\infty }\subset B_{M}$ be a sequence of connected submanifolds satisfying the following properties:

[M1] $x_{0}\in M_{n+1}\subset \mathrm{int}\left(M_{n}\right)$ for $n\in N_{0}$,
[M2] $f_{1}^{n_{0}}\left(M_{n+1}\right)\subset \mathrm{int}\left(M_{n}\right)$ for $n\in N_{0}$,
[M3] $\lim_{n\to \infty }\mathrm{diam}\left(M_{n}\right)=0$,
[M4] the sequence ${\left\{M_{n}\right\}}_{n=0}^{\infty }$ has the extension property.

Without loss of generality, we can also assume that $M_{0}\subset B\left(x_{0},\frac{\varepsilon }{3}\right)$. Obviously, there exists an open set U⊂M, such that $x_{0}\in U$ and $f_{1}^{n_{0}}\left(U\right)\subset B\left(x_{0},\frac{\varepsilon }{3}\right)$. Moreover, Condition [M3] implies that there exists $k^{*}>1$ such that $M_{k}\subset U$ for $k\geq k^{*}$.

Put $k_{1}=k^{*}+1$. Since $f_{1}^{n_{0}}$ is irreducible at $x_{0}\in \mathrm{int}\left(M_{k_{1}}\right)$, it is easy to see that there exist $x_{1}\in M_{k_{1}}$ and an arc $A(x_{0},f_{1}^{n_{0}}\left(x_{1}\right))$ with endpoints at $x_{0}$ and $f(x_{1})$ such that $A\left(x_{0},f_{1}^{n_{0}}\left(x_{1}\right)\right)\subset f_{1}^{n_{0}}\left(M_{k_{1}}\right)$. Let $A_{1}^{1},A_{2}^{1}$ be disjoint arcs such that $A_{1}^{1},A_{2}^{1}\subset A\left(x_{0},f_{1}^{n_{0}}\left(x_{1}\right)\right)$ and $x_{0}\notin A_{1}^{1}\cup A_{2}^{1}$. Put $\Gamma_{i}^{1}=f_{1}^{-n_{0}}\left(A_{i}^{1}\right)\cap M_{k_{1}}$ for i=1,2. Then, $\Gamma_{1}^{1}\neq \emptyset \neq \Gamma_{2}^{1}$, $x_{0}\notin \Gamma_{1}^{1}\cup \Gamma_{2}^{1}\subset M_{k_{1}}$, $\Gamma_{1}^{1}\cap \Gamma_{2}^{1}=\emptyset$ and the sets $\Gamma_{1}^{1}$ and $\Gamma_{2}^{1}$ are closed. Moreover, $f_{1}^{n_{0}}\left(\Gamma_{i}^{1}\right)=A_{i}^{1}$ for i=1,2.

On account of the well-known Hahn–Mazurkiewicz theorem (see, e.g., [19], p. 106), there exists a continuous function $g_{1}:A_{1}^{1}\cup A_{2}^{1}\to M_{k_{1}}$ such that $g_{1}\left(A_{1}^{1}\right)=M_{k_{1}}$ and $g_{1}\left(A_{2}^{1}\right)=M_{k_{1}}$. From the fact that the set $\Gamma_{1}^{1}\cup \Gamma_{2}^{1}$ is closed and from Condition [M3], it follows that there exists $k_{2}>k_{1}$ such that $M_{k_{2}}\cap \left(\Gamma_{1}^{1}\cup \Gamma_{2}^{1}\right)=\emptyset$. Obviously, $\left(A_{1}^{1}\cup A_{2}^{1}\right)\cap f_{1}^{n_{0}}\left(M_{k_{2}}\right)=\emptyset$.

By the same reasoning as above, one can find $x_{2}\in M_{k_{2}}$ and $A\left(x_{0},f_{1}^{n_{0}}\left(x_{2}\right)\right)\subset f_{1}^{n_{0}}(M_{k_{2})}$. Let $A_{1}^{2},A_{2}^{2},A_{3}^{2},A_{4}^{2}$ be such arcs that $A_{1}^{2}\cup A_{2}^{2}\cup A_{3}^{2}\cup A_{4}^{2}\subset A\left(x_{0},f_{1}^{n_{0}}\left(x_{2}\right)\right)$, $A_{i}^{2}\cap A_{j}^{2}=\emptyset$ if i≠j, $x_{0}\notin A_{1}^{2}\cup A_{2}^{2}\cup A_{3}^{2}\cup A_{4}^{2}$. Put $\Gamma_{i}^{2}=f_{1}^{-n_{0}}\left(A_{i}^{2}\right)\cap M_{k_{2}}$, i=1,⋯,4. Clearly $\overset{4}{\underset{i=1}{⋃}}\Gamma_{i}^{2}\subset M_{k_{2}}$, $\Gamma_{i}^{2}\cap \Gamma_{j}^{2}=\emptyset$ whenever i≠j, $x_{0}\notin \overset{4}{\underset{i=1}{⋃}}\Gamma_{i}^{2}$ and $\Gamma_{i}^{2}$ are closed for i=1,⋯,4. Moreover, $f_{1}^{n_{0}}\left(\Gamma_{i}^{2}\right)=A_{i}^{2}$ for i=1,⋯,4. Let $g_{2}:A_{1}^{2}\cup A_{2}^{2}\cup A_{3}^{2}\cup A_{4}^{2}\to M_{k_{2}}$ be a continuous function such that $g_{2}\left(A_{i}^{2}\right)=M_{k_{2}}$ for i=1,⋯,4.

Continuing in this fashion, we obtain two sequences: ${\left\{k_{i}\right\}}_{i\in N}\subset N$ and ${\left\{\Gamma_{i}\right\}}_{i\in N}$ of closed sets such that $\Gamma_{i}=\overset{2^{i}}{\underset{s=1}{⋃}}\Gamma_{s}^{i}\subset M_{k_{i}}$ for i∈N, $\Gamma_{s}^{i}$ is closed for $i\in N,s\in \{1,\cdots ,2^{i}\}$ and $\Gamma_{s_{1}}^{i}\cap \Gamma_{s_{2}}^{i}=\emptyset$ whenever $s_{1}\neq s_{2}$. Moreover, there exists a sequence ${\left\{g_{i}\right\}}_{i\in N}$ of continuous functions such that $g_{i}\left(f_{1}^{n_{0}}\left(\Gamma_{s}^{i}\right)\right)=M_{k_{i}}$ for $i\in N,s\in \{1,\cdots ,2^{i}\}$.

Now, let us consider the set $\Gamma =\overset{\infty }{\underset{i=1}{⋃}}\Gamma_{i}\cup \left\{x_{0}\right\}$. It follows easily that $\Gamma \subset \mathrm{int}(M_{1})$. It is easy to prove that Γ is closed.

Consider the following function:

$$g_{0}\left(x\right)=\left\{\begin{array}{cc} x_{0} & \;\mathrm{for}\;x=x_{0},\\ g_{i}\left(x\right) & \;\mathrm{for}\;x\in \overset{2^{i}}{\underset{s=1}{⋃}}\Gamma_{s}^{i},i\in N,\\ f_{1}^{n_{0}}\left(x\right) & \;\mathrm{for}\;x\in \mathrm{Fr}M_{k^{*}}. \end{array}\right.$$

Clearly, $g_{0}:\Gamma \cup \mathrm{Fr}M_{k^{*}}\to M_{k^{*}-1}$. Since $\Gamma \cup \mathrm{Fr}M_{k^{*}}$ is closed and $g_{0}$ is continuous, it follows by Condition [M4] that there exists a continuous function $g_{0}^{*}:M_{k^{*}}\to M_{k^{*}-1}$ such that $g_{0}^{*}↾\left(\Gamma \cup \mathrm{Fr}M_{k^{*}}\right)=g_{0}$.

Put:

$$\psi \left(x\right)=\left\{\begin{array}{cc} g_{0}^{*}\left(x\right) & \;\mathrm{for}\;x\in M_{k^{*}},\\ f_{1}^{n_{0}}\left(x\right) & \;\mathrm{for}\;x\notin M_{k^{*}}. \end{array}\right.$$

Consider the system:

$$T_{1,\infty }=\left\{f_{1},f_{2},\cdots ,f_{n_{0}},\psi ,f_{1},f_{2},\cdots ,f_{n_{0}},\psi ,\cdots \right\}.$$

We will show that $T_{1,\infty }$ is a periodic ε-disturbance of $f_{1,\infty }$. Condition (PD1) is obvious. To obtain Condition (PD2), it is enough to show that $\psi \in {\left[f_{1}^{n_{0}}\right]}_{x_{0}}^{\varepsilon }$. We have $\neq \left(\psi ,f_{1}^{n_{0}}\right)\subset M_{k^{*}}\subset B\left(x_{0},\varepsilon \right)$ because $\psi \left(x\right)=f_{1}^{n_{0}}\left(x\right)$ for $x\notin M_{k^{*}}$. Moreover, $f_{1}^{n_{0}}\left(\neq \left(\psi ,f_{1}^{n_{0}}\right)\right)\subset f_{1}^{n_{0}}\left(M_{k^{*}}\right)\subset f_{1}^{n_{0}}\left(U\right)\subset B\left(x_{0},\varepsilon \right)$ and $\psi \left(\neq \left(\psi ,f_{1}^{n_{0}}\right)\right)\subset \psi \left(M_{k^{*}}\right)=g_{0}^{*}\left(M_{k^{*}}\right)\subset M_{k^{*}-1}\subset B\left(x_{0},\varepsilon \right)$. This means that $\psi \frac{\varepsilon }{x_{0}}f_{1}^{n_{0}}$.

What is left is to prove that $x_{0}$ is an asymptotical A-focal entropy point of $T_{1,\infty }$.

Let *V* be an arbitrary open neighborhood of $x_{0}$. Obviously, there exists $k_{0}\in N$ such that $M_{k}\subset V$ for $k>k_{0}$. Let α∈R and α>0. We will show that there exists $A=\left(A_{1},\cdots ,A_{m}\right)\in \Theta \left(A\right)$ such that $H_{T_{1,\infty }^{n_{0}+1}}\left(A\right)\geq \left(n_{0}+1\right)\alpha .$ Obviously, one can find $i^{*}\in N$ such that $k_{i^{*}}>k_{0}$ and $i^{*}>\left(n_{0}+1\right)\alpha$. Thus, $\Gamma_{i^{*}}=\overset{2^{i^{*}}}{\underset{s=1}{⋃}}\Gamma_{s}^{i^{*}}\subset V$. Consider $A=(\Gamma_{1}^{i^{*}},\cdots ,\Gamma_{2^{i^{*}}}^{i^{*}})\subset V$, and put $\tilde{\psi }=\psi \circ f_{n_{0}}\circ \cdots \circ f_{1}$. Clearly, $T_{1,\infty }^{n_{0}+1}=\left(\tilde{\psi }\right)$.

Let k∈N. It is evident that $\mathrm{tr}(M_{T_{1,\infty }^{n_{0}+1}}^{k}\left(A\right))$ is equal to the number of (k+1)-paths connected with A. We have $\tilde{\psi }\left(\Gamma_{s}^{i^{*}}\right)=\psi \left(f_{1}^{n_{0}}\left(\Gamma_{s}^{i^{*}}\right)\right)=M_{k_{i^{*}}}$ and $\Gamma_{s}^{i^{*}}\subset M_{k_{i^{*}}}$ for any $s\in \{1,\cdots ,2^{i^{*}}\}$, so $\Gamma_{s_{1}}^{i^{*}}\underset{\tilde{\psi }}{⟶}\Gamma_{s_{2}}^{i^{*}}$ for $s_{1},s_{2}\in \left\{1,\cdots ,2^{i^{*}}\right\}$. As a consequence $\mathrm{tr}\left(M_{T_{1,\infty }^{n_{0}+1}}^{k}\left(A\right)\right)={\left(2^{i^{*}}\right)}^{k}$. Thus, $H_{T_{1,\infty }^{n_{0}+1}}\left(A\right)=\log2^{i^{*}}=i^{*}>\left(n_{0}+1\right)\alpha$.

Finally, we have shown that for any α>0, there exists A∈Θ(A), A⊂V such that $\frac{1}{n_{0}+1}H_{T_{1,\infty }^{n_{0}+1}}\left(A\right)>\alpha$. Hence for any α>0, we have $H\left(A,T_{1,\infty },V\right)=\sup\left\{\frac{1}{n}H_{T_{1,\infty }^{n}}\left(A\right):A\in \Theta \left(A\right)\wedge A\subset V\wedge n\in N\right\}\geq \sup\left\{\frac{1}{n_{0}+1}H_{T_{1,\infty }^{n_{0}+1}}\left(A\right):A\in \Theta \left(A\right)\wedge A\subset V\right\}\geq \alpha$. Thus, $H(A,T_{1,\infty },V)=+\infty$, and therefore, $d^{*}\left(A,T_{1,\infty },V\right)=1$, so $x_{0}$ is an asymptotical A-focal entropy point of $T_{1,\infty }$. ☐

The next theorem shows the difference between a A-focal entropy point of NDS and an asymptotical A-focal entropy point of NDS on the interval under as weak as possible assumptions imposed on the considered functions. For the simplicity of the notation, we will formulate and prove the theorem for $x_{0}=0$. It can be easily generalized for any $x_{0}\in \left[0,1\right]$.

**Theorem** **4.** *Let f:[0,1]→[0,1] be a function continuous at 0∈FIX(f) and such that h(f)<∞. Let us assume that:*

(*) *there exists a sequence $\alpha_{n}↘0$ such that for any n∈N, we have $f\left(\left[\alpha_{n},1\right]\right)\subset \left[\alpha_{n},1\right]$.*

*Then, for any ε>0, there exists a sequence ${\left\{f_{n}\right\}}_{n\in N}$ of functions continuous at zero such that ${\left\{f_{n}\right\}}_{n\in N}\subset {\left[f\right]}_{x_{0}}^{\varepsilon }$ and zero is a A-focal entropy point of the system $f_{1,\infty }$ and is not an asymptotical A-focal entropy point of $f_{1,\infty }$.*

**Proof.**  Let ε>0. Let γ be a positive number less than ε and such that f(x)<ε for x∈[0,γ]. There exists $n_{0}\in N$ such that $\alpha_{n_{0}}\in \left(0,\gamma \right)$ and $f\left(\left[\alpha_{n_{0}},1\right]\right)\subset \left[\alpha_{n_{0}},1\right]$. Put $\delta =\alpha_{n_{0}}$, and hence, f(δ)≥δ. Let m∈N be an odd positive integer such that logm>h(f).

From (*), it follows that there exists an interval P⊂(0,δ) such that f([δ,1])∩P=∅. Put $a_{0}=\inf P$ and $b_{0}=\sup P$. Notice that $0<b_{0}<\delta$. Consider a sequence $x_{n}↘0$ such that $x_{1}=a_{0}$. Now, we can define the function $f_{1}:\left[0,1\right]\to \left[0,1\right]$ as follows: $f_{1}\left(0\right)=0$, $f_{1}\left(x_{n+1}+2k\frac{x_{n}-x_{n+1}}{m}\right)=x_{n+1}$ for $k\in \{0,1,\cdots ,\frac{m-1}{2}\}$, $f_{1}\left(x_{n+1}+\left(2k-1\right)\frac{x_{n}-x_{n+1}}{m}\right)=x_{n}$ for $k\in \{1,\cdots ,\frac{m+1}{2}\}$, $f_{1}\left(x_{n+1}+\frac{x_{n}-x_{n+1}}{2m}\right)=x_{n+1}+\frac{x_{n}-x_{n+1}}{2m}$, $f_{1}\left(x_{n}-\frac{x_{n}-x_{n+1}}{2m}\right)=x_{n}-\frac{x_{n}-x_{n+1}}{2m}$, $f_{1}$ is linear on respective intervals in each $\left[x_{n+1},x_{n}\right]$; and moreover, $f_{1}\left(x\right)=a_{0}$ for $x\in [a_{0},b_{0})$, $f_{1}\left(x\right)=b_{0}$ for $x\in [b_{0},\delta )$ and $f_{1}\left(x\right)=f\left(x\right)$ for x∈[δ,1].

We next define functions $f_{n}$ for n≥2. Let $f_{n}\left(x\right)=f_{1}\left(x\right)$ for $x\in \left[0,1\right]\backslash \left(a_{0},\delta \right)$, n≥2. Fix $y_{0}\in \left(a_{0},b_{0}\right)$. Put $f_{n}\left(a_{0}+2k\frac{y_{0}-a_{0}}{m+2}\right)=a_{0}$ for $k\in \{0,1,\cdots ,\frac{m+1}{2}\}$ and $f_{n}\left(a_{0}+\left(2k-1\right)\frac{y_{0}-a_{0}}{m+2}\right)=y_{0}$ for $k\in \{1,\cdots ,\frac{m+3}{2}\}$ and $f_{n}$ linear on the respective intervals. Moreover, $f_{n}\left(x\right)=y_{0}$ for $x\in [y_{0},b_{0})$, $f_{n}\left(x\right)=b_{0}$ for $x\in [b_{0},\delta )$.

Obviously $f_{n}$ is continuous at zero for n∈N and ${\left\{f_{n}\right\}}_{n\in N}\subset {\left[f\right]}_{0}^{\varepsilon }$. We will show that $h\left(f_{1}\right)=\log m=h\left(f_{1,\infty }\right)$ and $h\left(f_{n}\right)=\log\left(m+2\right)=h\left(f_{n,\infty }\right)$ for n≥2.

We first prove that $h(f_{1},\left[a_{0},b_{0}\right))=0$. Let $\varepsilon_{1}>0$, n∈N and $M\subset [a_{0},b_{0})$ be an $\left(n,\varepsilon_{1}\right)$-separated set for $f_{1}$. For any x,y∈M, x≠y, there exists $i_{0}\in \left\{0,\cdots ,n-1\right\}$ such that $\rho \left({\left(f_{1}\right)}^{i_{0}}\left(x\right),{\left(f_{1}\right)}^{i_{0}}\left(y\right)\right)>\varepsilon_{1}$. Notice that $i_{0}=0$. Indeed, we have $f_{1}\left(x\right)=a_{0}$ and $f_{1}\left(y\right)=a_{0}$. Hence, for i>0, we have ${\left(f_{1}\right)}^{i}\left(x\right)=a_{0}$ and ${\left(f_{1}\right)}^{i}\left(y\right)=a_{0}$, so $\rho ({\left(f_{1}\right)}^{i}\left(x\right),{\left(f_{1}\right)}^{i}\left(y\right))=0$ for i>0. As a consequence, for any distinct points x,y∈M, we have $\rho \left(x,y\right)>\varepsilon_{1}$. It follows that $\#\left(M\right)\leq \left[\frac{b_{0}-a_{0}}{\varepsilon_{1}}\right]+1$, so $s_{n}\left(f_{1},\left[a_{0},b_{0}\right),\varepsilon_{1}\right)\leq \left[\frac{b_{0}-a_{0}}{\varepsilon_{1}}\right]+1$, where $\left[\frac{b_{0}-a_{0}}{\varepsilon_{1}}\right]$ denotes the smallest positive integer greater than $\frac{b_{0}-a_{0}}{\varepsilon_{1}}$. Hence, $h\left(f_{1},\left[a_{0},b_{0}\right)\right)=\lim_{\varepsilon_{1}\to 0}\underset{k\to \infty }{lim sup}\frac{1}{k}\log\left(s_{k}\left(f_{1},\left[a_{0},b_{0}\right),\varepsilon_{1}\right)\right)\leq 0$. In an analogous way, one can show that $h(f_{1},\left[b_{0},\delta \right))=0$.

Moreover, we have $f_{1}\left(x\right)=f\left(x\right)$ for x∈[δ,1] and $f_{1}\left(\left[\delta ,1\right]\right)\subset \left[\delta ,1\right]$. Consequently, $h\left(f_{1},\left[\delta ,1\right]\right)=h\left(f,\left[\delta ,1\right]\right)<\log m$.

Let n∈N. We will show that $h(f_{1},\left[x_{n+1},x_{n}\right])=\log m$. Clearly, $f_{1}↾\left[x_{n+1},x_{n}\right]:\left[x_{n+1},x_{n}\right]\to \left[x_{n+1},x_{n}\right]$ and $f_{1}↾\left[x_{n+1},x_{n}\right]$ is piecewise monotone. Denote by $c_{k}$ the number of intervals of monotonicity of ${\left(f_{1}\right)}^{k}$. We have $c_{k}=m^{k}$ for k∈N. Thus, by Theorem 4.2.4 [20], we have $h\left(f_{1},\left[x_{n+1},x_{n}\right]\right)=\lim_{k\to \infty }\frac{1}{k}\log c_{k}=\log m$. Obviously, $\left[0,a_{0}\right]=\underset{n\in N}{⋃}\left[x_{n+1},x_{n}\right]$, and for any n∈N, we have $f_{1}\left(\left[x_{n+1},x_{n}\right]\right)\subset \left[x_{n+1},x_{n}\right]$. On account of Lemma 4.1.10 [20] (and the remark after it), we obtain $h\left(f_{1},\left[0,a_{0}\right]\right)=\underset{n\in N}{\sup}\left(h\left(f_{1},\left[x_{n+1},x_{n}\right]\right)\right)=\log m$. Finally, Proposition 3.5 [12] (see also Lemma 4.1 from [13]) gives that $h\left(f_{1}\right)=\max\left\{h\left(f_{1},\left[0,a_{0}\right]\right),h\left(f_{1},\left[a_{0},b_{0}\right)\right),h\left(f_{1},\left[b_{0},\delta \right)\right),h\left(f_{1},\left[\delta ,1\right]\right)\right\}=\log m$.

We now turn to the case n≥2. We have $f_{n}↾\left[0,a_{0}\right]=f_{1}↾\left[0,a_{0}\right]$ and $f_{1}:\left[0,a_{0}\right]\to \left[0,a_{0}\right]$. Hence, $h\left(f_{n},\left[0,a_{0}\right]\right)=h\left(f_{1},\left[0,a_{0}\right]\right)=\log m$. Moreover, $f_{n}↾\left[\delta ,1\right]=f_{1}↾\left[\delta ,1\right]$ and $f_{1}:\left[\delta ,1\right]\to \left[\delta ,1\right]$, so $h\left(f_{n},\left[\delta ,1\right]\right)=h\left(f_{1},\left[\delta ,1\right]\right)<\log m$. Therefore, $f_{n}↾\left[b_{0},\delta \right)=f_{1}↾\left[b_{0},\delta \right)$ and $f_{1}:\left[b_{0},\delta \right)\to \left[b_{0},\delta \right)$, so $h\left(f_{n},\left[b_{0},\delta \right)\right)=h\left(f_{1},\left[b_{0},\delta \right)\right)=0$.

As was done for a function $f_{1}$, one can show that $h(f_{n},\left[y_{0},b_{0}\right))=0$ and $h\left(f_{n},\left[a_{0},y_{0}\right]\right)=\log\left(m+2\right)$. As a consequence, by Proposition 3.5 [12], we obtain $h\left(f_{n}\right)=\log\left(m+2\right)$.

Since $f_{n}=f_{2}$ for n≥2, it follows that $h\left(f_{n,\infty }\right)=h\left(f_{2}\right)=\log\left(m+2\right)$ for n≥2.

We will show now that $h(f_{1,\infty })=\log m$. We claim that:

(10) $${\left(f_{1}\right)}^{i}\left(z\right)=f_{1}^{i}\left(z\right)\;\mathrm{for}\;z\in \left[0,1\right]\;\mathrm{and}\;i\in N_{0}.$$

Indeed, if $z\in (a_{0},b_{0})$, then $f_{1}\left(z\right)=a_{0}$. Thus, for i≥1, we have ${\left(f_{1}\right)}^{i}\left(z\right)=a_{0}$ and $f_{1}^{i}\left(z\right)=a_{0}$, so ${\left(f_{1}\right)}^{i}\left(z\right)=f_{1}^{i}\left(z\right)$. For i=0, we have ${\left(f_{1}\right)}^{0}\left(z\right)=z=f_{1}^{0}\left(z\right)$, so ${\left(f_{1}\right)}^{i}\left(z\right)=f_{1}^{i}\left(z\right)$ for $z\in (a_{0},b_{0})$ and $i\in N_{0}$. If $z\in \left[0,1\right]\backslash \left(a_{0},b_{0}\right)$ then $f_{n}\left(z\right)\in \left[0,1\right]\backslash \left(a_{0},b_{0}\right)$ for n∈N. Therefore, it is easy to see that for $z\in \left[0,1\right]\backslash \left(a_{0},b_{0}\right)$ and i≥1, we have ${\left(f_{1}\right)}^{i}\left(z\right)=f_{1}^{i}\left(z\right)$. Obviously, ${\left(f_{1}\right)}^{0}\left(z\right)=z=f_{1}^{0}\left(z\right)$. The proof of (10) is complete.

Notice that for any n∈N and $\varepsilon_{1}>0$, the set M⊂[0,1] is $\left(n,\varepsilon_{1}\right)$-separated for the system $f_{1,\infty }$ if and only if *M* is $\left(n,\varepsilon_{1}\right)$-separated for $f_{1}$. Indeed, let *M* be an $\left(n,\varepsilon_{1}\right)$-separated set for $f_{1}$. Then, for any distinct points x,y∈M, there exists i∈{0,⋯,n−1} such that $\rho \left({\left(f_{1}\right)}^{i}\left(x\right),{\left(f_{1}\right)}^{i}\left(y\right)\right)>\varepsilon_{1}$. By (10), we obtain $\rho \left(f_{1}^{i}\left(x\right),f_{1}^{i}\left(y\right)\right)>\varepsilon_{1}$, which means that *M* is an $\left(n,\varepsilon_{1}\right)$-separated set for the system $f_{1,\infty }$. The proof of the converse implication runs in a similar way.

As a consequence, we have $s_{n}\left(f_{1},\left[0,1\right],\varepsilon_{1}\right)=s_{n}\left(f_{1,\infty },\left[0,1\right],\varepsilon_{1}\right)$, so $\log m=h\left(f_{1}\right)=h\left(f_{1,\infty }\right)$.

Let *U* be an arbitrary neighborhood of zero. We will show that $H(A,f_{1,\infty },U)=\log m.$ Clearly, by Theorem 1, we have:

(11) $$H\left(A,f_{1,\infty },U\right)\leq h\left(f_{1,\infty }\right)=\log m.$$

Let n∈N. Consider the interval $\left[x_{n+1},x_{n}\right]$. There exists a sequence of points $x_{n+1}<a_{n,1}<b_{n,1}<a_{n,2}<b_{n,2}<\cdots <a_{n,m}<b_{n,m}<x_{n}$ such that $f_{1}\left(\left[a_{n,i},b_{n,i}\right]\right)=\left[a_{n,i},b_{n,i}\right]$ for i∈{1,⋯,m}. Put $A_{i}^{n}=\left[a_{n,i},b_{n,i}\right]$ for i∈{1,⋯,m}. Then, $A^{n}=\left(A_{1}^{n},\cdots ,A_{m}^{n}\right)\in \Theta \left(A\right)$ and:

(12) $$\mathrm{for}\;\mathrm{any}\;k\in N\;\mathrm{and}\;\mathrm{any}\;i,j\in \left\{1,\cdots ,m\right\}\;\mathrm{we}\;\mathrm{have}\;A_{i}^{n}\underset{f_{k}}{\to }A_{j}^{n}.$$

Obviously, for any k∈N, the trace $\mathrm{tr}(M_{f_{1,\infty }}^{k}\left(A^{n}\right))$ is equal to the number of (k+1)-paths with the first and the last node at $A_{i}^{n}$ for i=1,⋯,m. By (12), we conclude that the number of such paths is equal to $m^{k}$. Hence, $\frac{1}{k}\log\mathrm{tr}\left(M_{f_{1,\infty }}^{k}\left(A^{n}\right)\right)=\log m$, and therefore:

(13) $$H_{f_{1,\infty }}\left(A^{n}\right)=\log m.$$

Let $n_{0}\in N$ be such that $[x_{n_{0}+1},x_{n_{0}}]\subset U$. Then, by (13), we obtain $H_{f_{1,\infty }}\left(A^{n_{0}}\right)=\log m$, so $H(A,f_{1,\infty },U)\geq \log m$. From this and (11), we get $H(A,f_{1,\infty },U)=\log m$. As a consequence, $d(A,f_{1,\infty },U)=1$, which gives $E(A,f_{1,\infty },0)=1$, so zero is a A-focal entropy point of $f_{1,\infty }$.

Simultaneously, zero is not an asymptotical A-focal entropy point of $f_{1,\infty }$, because for any neighborhood *U* of zero, we have $H(A,f_{1,\infty },U)=\log m$ and $h^{*}\left(f_{1,\infty }\right)=\lim_{n\to \infty }h\left(f_{n,\infty }\right)=\log\left(m+2\right)$. Therefore, $d^{*}\left(A,f_{1,\infty },U\right)=\frac{\log m}{\log(m+2)}$, which means that $E^{*}\left(A,f_{1,\infty },0\right)=\frac{\log m}{\log(m+2)}<1$. ☐

## 4. Conclusions

In the paper, the notions of a focal entropy point and an asymptotical focal entropy point for nonautonomous dynamical systems are introduced. The definitions adopted in the paper specify the notions that express the complexity of a system around these points and moreover, the complexity of a system around such points does not depend on the behavior of the system in other parts of its domain. Each asymptotical focal entropy point of an NDS is its focal entropy point. In the case of periodic dynamical systems these notions coincide. For a periodic NDS consisting of continuous functions defined on the closed unit interval there exists an asymptotical focal entropy point. Moreover, there exists a dynamical system with a focal entropy point which is not its asymptotical focal entropy point. In the case of some periodic dynamical systems consisting of continuous functions defined on a topological manifold one can disturb a system to obtain a system “lying close” to the given one and having an asymptotical focal entropy point.
